# Prominent hyperplasia of renin-producing juxtaglomerular apparatus after chronic and complete blockade of the renin-angiotensin system in adult IgA nephropathy

**DOI:** 10.1007/s13730-015-0177-y

**Published:** 2015-04-09

**Authors:** Michiaki Abe, Kensuke Joh, Norio Ieiri, Osamu Hotta, Yasunori Utsunomiya, Hiroshi Sato, Kiyomi Kisu, Naoki Sakumo, Hideyasu Kiyomoto, Toshinobu Sato, Yoshio Taguma, Sadayoshi Ito

**Affiliations:** 1 Department of Nephrology, Japan Community Health Care Organization Sendai Hospital, Sendai, Japan; 2 Department of Education and Support for Regional Medicine, Tohoku University Hospital, 1-1 Seiryo-cho, Aoba-ku, Sendai, 980-8574 Japan; 3 Division of Nephrology, Endocrinology and Vascular Medicine, Department of Medicine, Tohoku University Hospital, Sendai, Japan; 4 Department of Pathology, Tohoku University Graduate School of Medicine, Sendai, Japan; 5 Division of Nephrology, Department of Medicine, Tokyo Jikei University, Tokyo, Japan; 6 Hotta Osamu Clinic, Sendai, Japan

**Keywords:** IgA nephropathy, Renin-angiotensin system, Juxtaglomerular apparatus

## Abstract

Juxtaglomerular apparatus (JGA) hyperplasia rarely happened in renal biopsy and has been controversial clinically, because synthesis and secretion of renin were susceptible to the effect of clinical condition and medication. Here we present the case of a 39-year-old who got JGA hyperplasia of IgA nephropathy (IgAN) after long-term inhibition of the renin-angiotensin system (RAS) with an angiotensin receptor blocker (ARB), and a direct renin inhibitor (DRI) in combination with a diuretic. He was diagnosed with IgAN in his first renal biopsy, and was treated with supra-maximal dosages of ARB, DRI and a diuretic. In the second biopsy, because of the massive proteinuria and occurrence of steroid-induced diabetes, it was revealed that the area and the number of JGA cells were strikingly increased in observed glomeruli. Immunohistopathologically, the both specimens were stained by human renin antibody. The hyperplastic JG cells contained a large amount of renin granules. Putative renin granules were observed in some interstitial cells adjacent to an afferent arteriole by electron microscopy. The increasing response of renin granules co-localized in prominent JGA hyperplasia should be worried while physicians treat hypertensive patients with potent RAS inhibitors and diuretics even though they have diabetes. This is the first report showing a clinical course of forming prominent JGA hyperplasia directly after a full combination of RAS inhibitors and diuretics in adult IgA nephropathy.

## Introduction

Renin is a primary step of the renin-angiotensin system (RAS). It is critically linked to fluid volume, blood pressure and electrolyte homeostasis of the body. Renin is mainly produced and secreted by the juxtaglomerular apparatus (JGA) [1]. JGA hyperplasia is rarely recognized in renal biopsy specimens of common renal diseases but recognized in Bartter syndrome or prolonged extreme dehydration. In this paper, we report a case of prominent JGA hyperplasia developed in an IgA nephropathy (IgAN) patient after potent inhibition treatment of RAS under using diuretics.

## Materials and methods

### Case report

**Fig. 1:**
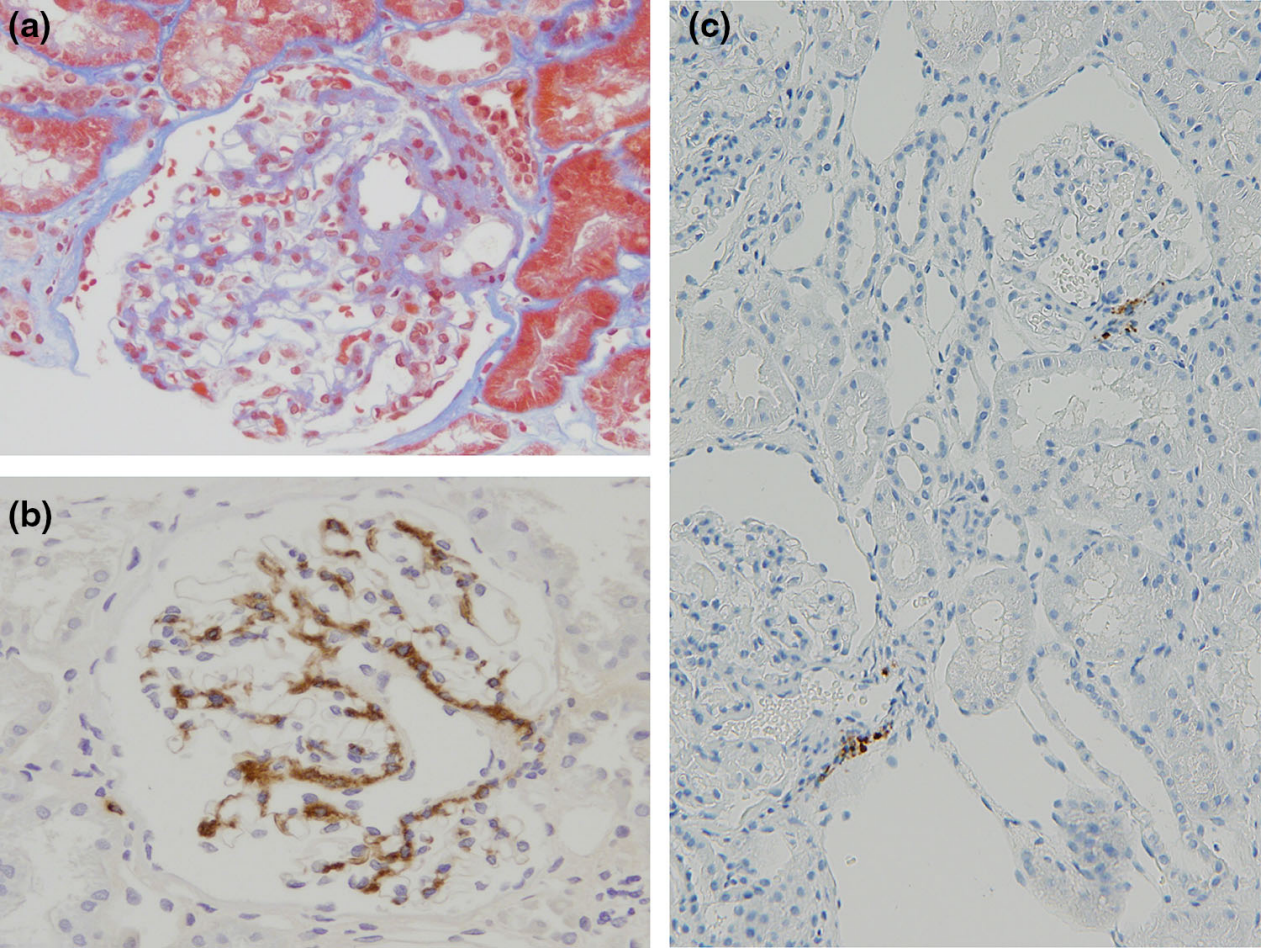
Renal histopathology of the first biopsy. **a** Masson stain and **b** immunoperoxidase staining for IgA. **c** Immunoperoxidase staining for renin

**Fig. 2:**
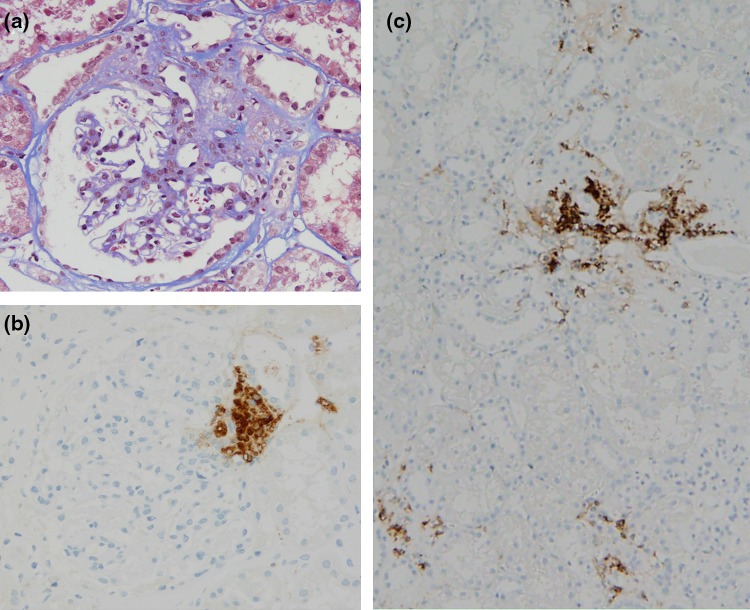
Renal histopathology of the second biopsy. **a** Masson stain, **b** immunoperoxidase staining for renin vesicles in the JGA and **c** the interstitium

**Fig. 3:**
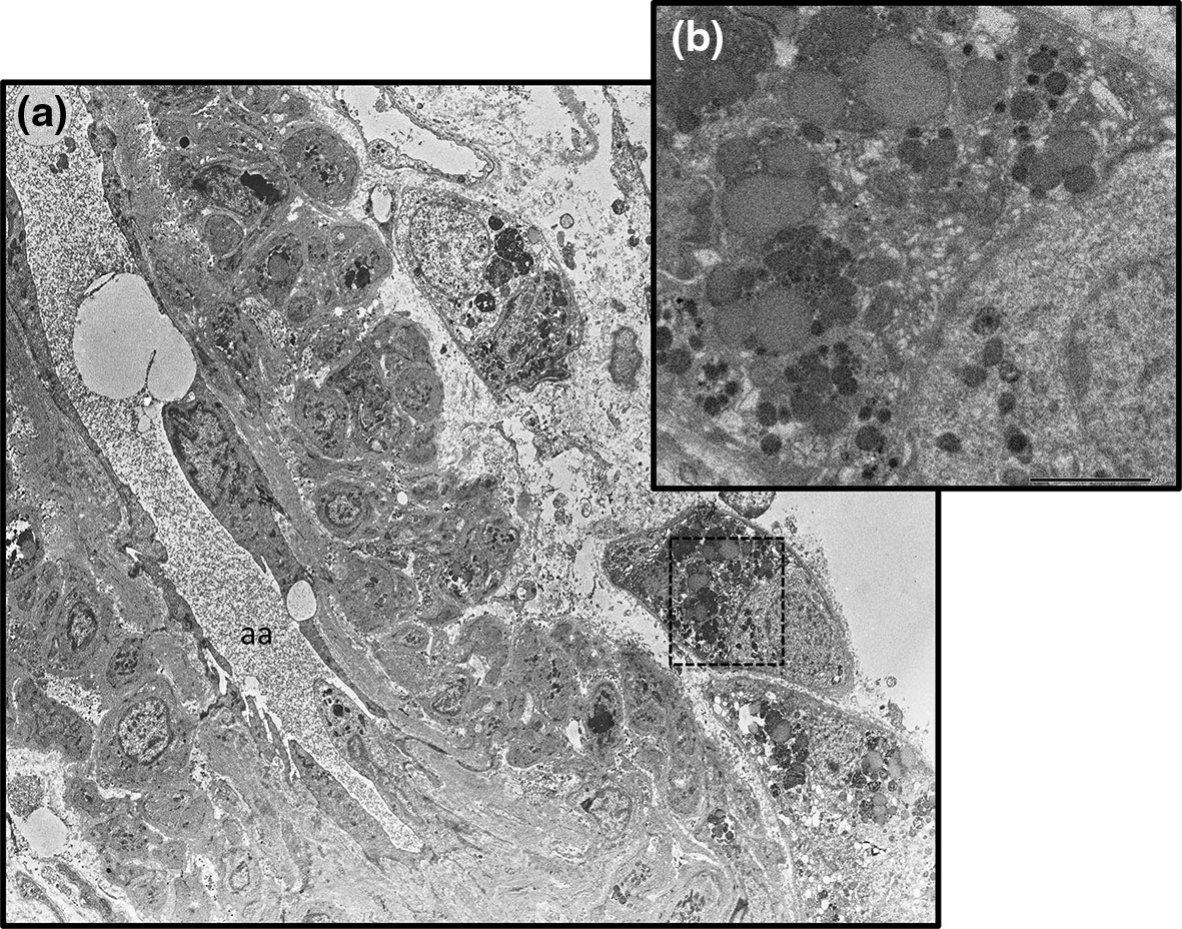
Electron microscopy. **a** Interstitial cells containing putative renin granules adjacent to tubular cells. **b** To observe putative renin granules well, a magnified picture of *dotted box* in **a** with *scale bar* (2.0 µm), and *aa* indicates afferent arteriole

Our subject, a 39-year-old businessman, has had a history of hematuria since 11 y.o. High blood pressure (BP) was pointed at 23 years but it was left untreated. When he was 30 y.o., his first renal biopsy was preformed because of proteinuria. His physical examination results were as follows: body height (BH) 184 cm, body weight (BW) 118 kg, BMI 34.9, BP 140/100 mmHg, serum creatinine (sCr) 0.5 mg/dL, creatinine clearance (CrCl) 131 mL/min, proteinuria (UP 0.2 g/day) and microscopic hematuria. Pathologically, his disease was diagnosed as IgAN (total of 12 glomeruli obtained: global sclerosis 7 %, IgA deposition 3+ diffuse/mesangial, C3 deposition 1+ diffuse/mesangial, Fig. 1a, b) without any active lesions (Oxford classification: M0, S0, E0, T0), and the JGA was not hyperplastic. To control his blood pressure, he had been treated with a daily dosage of losartan 100 mg, hydrochlorothiazide 12.5 mg, lisinopril 10 mg, amlodipine 5 mg, cilnidipine 5 mg, dipyridamole 300 mg and allopurinol 100 mg for 8 years. He had kept on the same medication and dietary therapy, but his renal function was chronically worsened with increases in his sCr 1.24 mg/dL, CrCl 90 mL/min, and UP 1.5 g/day at 38 years. He moved on business and his prescription medication was changed to a daily dosage of valsartan 320 mg and aliskiren 300 mg, hydrochlorothiazide 25 mg and allopurinol 100 mg. However, his proteinuria increased to 4.5 g/day, alternative-daily administration of 60 mg prednisolone (PSL) was started at 39 years. But the nephrotic range of proteinuria remained consistent, and he suffered from severe edema and general fatigue. The next month, he left the job for visiting our hospital and his physical examination were as follows; BH 184 cm, BW 109.4 kg, BMI 32.3, BP 122/74 mmHg, sCr 1.62 mg/dL, urea N 26 mg/dL, urate 9.4 mg/dL, sNa 138 mEq/L, sK 4.0 mEq/L, sCl 104 mEq/L, sCa 9.2 mg/dL, sIP 3.2 mg/dL, albumin 3.8 g/dL, HbA1c 6.4 %, CrCl 46.4 mL/min, UP 0.10–0.15 g/day, FENa 1.2 %. As steroid-induced diabetes mellitus was diagnosed as HbA1c 8.2 %, oral administration of PSL was reduced to 40 mg on alternate days. The second biopsy was performed again in September and revealed advanced IgAN compared to his first biopsy (M1, S0, Eo, T2; glomeruli 14, global sclerosis 43 %, IgA deposition 3+ diffuse/mesangial, IgM deposition 1+ diffuse/mesangial, C3 2+ diffue/mesangial, Fib+ diffuse/mesangial). In addition, we observed glomerular hyperplasia (max glomerular diameter 300 µm). Moreover, it was noteworthy that hyperplasia of JGA was observed in almost glomeruli observed in the second biopsy (Fig. 2a). To achieve IgAN remission, the subject chose tonsillectomy treatment and methylprednisolone semi-pulse therapy (500 mg, 3 days per week) for 3 weeks [2], and post-treatment with oral administration of PSL (30 mg alternate days). To control his blood pressure, both ARB and renin DRI were continued but the dosages were reduced (Fig. 3). Complete remission from hematuria and proteinuria (UP < 0.05 g/gCr) has been maintained for four years, even while PSL therapy was tapered by 5 mg/q.d. every 2 months and discontinued now.

The patient gave his informed consent for the case report that was approved by the Sendai-Shakaihoken-Hospital IRB/Ethics Committee, IRB approval number 2014-1.

### Immunohistochemistry

Immunohistochemical staining for renin was performed using anti-human renin antibody (1:500), which was kindly provided by Suzuki F., Gifu University.

## Results

When the anti-human renin antibody detected renin granules, the positive area was located only in normal JGA and not in interstitium (anti-human renin antibody was kindly provided by Suzuki, Gifu University [3]) as shown in Fig. 1c. An immunohistochemical examination clearly revealed that the JGA contained many renin granules (Fig. 2b). In addition, the interstitial cells contained renin granules (Fig. 2c). To observe renin granules better, we additionally examined electron microscopy analysis. Putative renin granules were observed in interstitial cells adjacent to an afferent arteriole (Fig. 3). Representative of renin granules were referred from a previous report [4]. But we could not identify them as extended JGA cells or not.

Because the occurrence of plasma active renin concentration (PRC) increased remarkably (440 pg/mL; reference range up to 2.5–21 pg/mL during supine position), plasma renin activity (PRA) and plasma aldosterone concentration (PAC) increased minimally (2.6 ng/mL/hr and 42.4 pg/mL, respectively).

## Discussion

The occurrence of JGA hyperplasia in an IgAN patient after prolonged complete RAS inhibition with ARB and DRI combined with thiazide diuretics is described. Although JGA looked normal in the first biopsy, areas of striking JGA hyperplasia containing increment of intracellular renin granules were ubiquitously recognized in the second biopsy. Previous report demonstrated renin-positive granules were observed in tubular epithelial cells, apparent atrophic and cell-rich glomeruli and walls of tortuous arterioles on reflux nephropathy [4]. Collecting duct was reported as a major source of prorenin in a diabetic animal model [5]. However, we could not find renin granules in tubular cells except for degrading granules like lysosomes in our electron microscopic analysis. In addition, we did not determine type of the cells, which is a limitation in the case study. These unusual characteristics of the renal biopsy have presumably developed in response to his clinical condition and/or his medical treatments. Moreover, PRC increased noticeably compared with PRA under DRI treatment. The abroad global glomerular sclerosis probably caused by hypertension and obesity, as well as long-term active stage of IgAN.

It is a well-known fact that the RAS inhibitor is effective in IgAN [6, 7] because of improvement in intraglomerular hypertension as to dilate more efferent arteriole than afferent arteriole. Nishiyama et al. [8] also reported that urinary angiotensinogen reflects intrarenal angiotensin II, which is higher in IgAN than those in minor glomerular abnormalities. Interestingly, Nakanishi et al. [9] showed that long-term administration of ARBs induces an extreme increase of renin-producing cells and unusual proliferation of smooth muscle cells in afferent arteriolar walls in obese and diabetic rats. Their results were similar to changes as we observed clinically in this case.

**Fig. 4:**
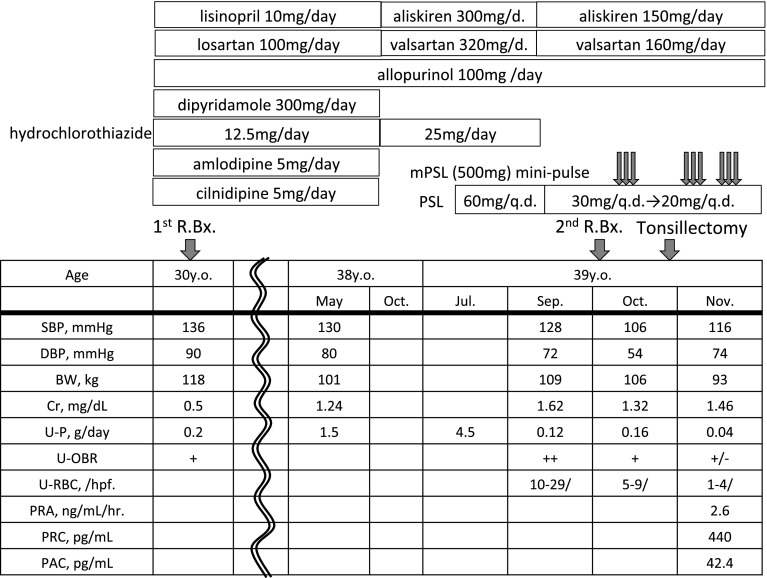
Clinical coarse around the first and second renal biopsy. *R.Bx.* renal biopsy, *q./d.* alternated-date, *SBP* systolic blood pressure, *DBP* diastolic blood pressure, *BW* body weight, *Cr* creatinine, *U*-*P* urinary proteinuria, *U*-*OBR* urinary occult blood reaction, *U*-*RBC* urinary red blood cell, *hpf.* high power field

Renin synthesis is stimulated by the cyclic adenosine monophosphate (cAMP) pathway [10] due to β1-adrenergic receptor activation, prostaglandins, nitric oxide and cAMP-phosphodiesterases inhibitors. Physiologically, renin secretion is increased by RAS inhibitors, macula densa control and activation of the renal baroreceptor mechanism with salt-depletion, dehydration and/or ischemia [11]. In this case (clinical course in Fig. 4), the blood pressure was high and there was no symptom of dehydration, and the blood glucose was well controlled on admission for the second biopsy. In addition, the patient did not take any β1-adrenergic blockers, NSAIDs and theophylline. We suspected that the development of JGA hyperplasia was mainly due to complete inhibition of the RAS and partially diuretics.

Clinically, hyperreninemia is well recognized in salt-depletive state on Batter syndrome, pseudo-Batter syndrome or during diuretics as well as hypertensive state of renovascular hypertension (long-loop feedback). In addition, RAS inhibitors stimulated renin synthesis (short-loop feedback). There is no relationship between hyper-secretion of renin and high blood pressure as shown in previous study [12]. RAS inhibition modified renin and blood pressure of hypertensive patients. Here, there are hypertension and hyper-secretion of renin, but not high concentration of aldosterone and hypokalemia. This case was neither Batter syndrome nor pseudo-Batter syndrome, and renal arterial stenosis was denied by an ultrasonography of renal artery. Presumably, the diuretic is considered to benefit for an unstable salt intake in this case. Once JGA apparatus happened, hyperplasia due to potent RAS inhibition and diuretics, pathological change of JGA hyperplasia is not quickly shrunk after breaking the treatments. So, sudden break of the treatments could lead to hypertension by activation of hyperreninemic state and relatively excess salt intake. Continuation of salt reduction is also notable in these patients.

In conclusion, JGA hyperplasia is occurred by both long-loop feedback of renin synthesis by salt depletion state or short-feedback by multiple RAS inhibitors. We note that intense use of RAS inhibitors in combination with diuretics in IgAN patients happens to develop JGA hyperplasia by both long and short-feedback like this case, even if there were no symptoms of dehydration and hypotension.
